# WAVE3 phosphorylation regulates the interplay between PI3K, TGF-β, and EGF signaling pathways in breast cancer

**DOI:** 10.1038/s41389-020-00272-0

**Published:** 2020-10-05

**Authors:** Wei Wang, Urna Kansakar, Vesna Markovic, Bingcheng Wang, Khalid Sossey-Alaoui

**Affiliations:** 1 Department of Medicine, Rammelkamp Center for Research, Cleveland, OH USA; 2 Case Western Reserve University School of Medicine, Cleveland, OH USA; 3 Case Comprehensive Cancer Center, Cleveland, OH USA

**Keywords:** Growth factor signalling, Breast cancer, Extracellular signalling molecules, Cancer genetics

## Abstract

Both TGF-β and the PI3K-AKT signaling pathways are known activators of various intracellular pathways that regulate critical cellular functions, including cancer cell survival and proliferation. The interplay between these two oncogenic pathways plays a major role in promoting the initiation, growth, and progression of tumors, including breast cancers. The molecular underpinning of the inter-relationship between these pathways is, however, not fully understood, as is the role of WAVE3 phosphorylation in the regulation of tumor growth and progression. WAVE3 has been established as a major driver of the invasion–metastasis cascade in breast cancer and other tumors of epithelial origin. WAVE3 phosphorylation downstream of PI3K was also shown to regulate cell migration. Here we show that, in addition to PI3K, WAVE3 tyrosine phosphorylation can also be achieved downstream of TGF-β and EGF and that WAVE3 tyrosine phosphorylation is required for its oncogenic activity. Our in vitro analyses found loss of WAVE3 phosphorylation to significantly inhibit cell migration, as well as tumorsphere growth and invasion. In mouse models for breast cancer, loss of WAVE3 phosphorylation inhibited tumor growth of two aggressive breast cancer cell lines of triple-negative subtype. More importantly, we found that WAVE3 phosphorylation is also required for the activation of PI3K, TGF-β, and EGF signaling and their respective downstream effectors. Therefore, our study identified a novel function for WAVE3 in the regulation of breast cancer development and progression through the modulation of a positive feedback loop between WAVE3 and PI3K-TGF-β-EGF signaling pathways.

## Introduction

Breast cancer (BC) is the second leading cause of cancer-related deaths in women^1^. A myriad of molecular signaling pathways are dysregulated in BC tumors and contribute to their aggressiveness. Phosphatidylinositol-3 kinase (PI3K)/AKT, transforming growth factor-β (TGF-β)/SMAD, and mitogen-activated protein kinase/extracellular signal-regulated kinase 1/2 (ERK1/2) are well-established oncogenic pathways in several cancers, including BC^2^. Mutations in PI3K catalytic subunit alpha, PI3KCA, are found in >50% of breast tumors^3,4^. In the triple-negative breast cancer (TNBC) subtype, which is considered the most aggressive of all BC subtypes, PI3KCA mutation rates are as high as 16%^3,5^. TGF-β plays a tumor-suppressor role in early stages of BC, and its function suddenly converts to a tumor promoter in late-stage BC, hence the TGF-β paradox^6^. In the TGF-β signaling, SMAD is the best-known canonical downstream effector^7,8^. However, several non-SMAD (non-canonical TGF-β) pathways have also been implicated in mediating the oncogenic activities of TGF-β, including the PI3K and the ERK^9–13^. In BC, TGF-β induces phosphorylation and activation of the PI3K-dependent AKT serine–threonine kinase activity^14–17^, resulting in the induction of epithelial-to-mesenchymal transition (EMT), migration, and invasion of cancer cells, all of which are hallmarks of cancer progression and metastasis^18,19^. On the other hand, both TGF-β and PI3K activate ERK1/2 and its downstream effectors to stimulate cell survival and anti-apoptotic pathways in cancer^12,20–22^. The complexity of interactions between these oncogenic pathways are poorly understood, prompting the need for investigations of the molecular mechanisms involved in such complex interrelationships that ultimately lead to the aggressive behavior of specific BC subtypes, such as TNBCs.

WAVE3 is a member of the WASP/WAVE family of actin-cytoskeleton remodeling proteins^23–26^. WAVE3 plays an essential role in the regulation of cancer cell migration and invasion^27–30^. WAVE3 have been established as a major driver of the invasion–metastasis cascade in BC by regulating EMT^31–33^. The oncogenic activity of WAVE3 is also driven by its regulation the cancer stem cell (CSC) niche in BC^34,35^. We and others previously reported that WAVE3 can be phosphorylated, downstream of PI3K at four tyrosine residues, leading to activation of cell migration^27,35–38^. Whether WAVE3 phosphorylation plays a major role in the regulation of BC development and progression is, however, not known, as is the role of WAVE3 phosphorylation in the regulation of PI3K and TGF-β signaling. Here we show that, in addition to PI3K, WAVE3 phosphorylation can be achieved downstream of TGF-β and epidermal growth factor (EGF). We also show that WAVE3 phosphorylation is required for cancer cell migration, invasion, and BC tumor growth. More importantly, we found inhibition of WAVE3 phosphorylation to negatively affect the downstream signaling effectors of PI3K, TGF-β, and EGF, therefore, identifying a new feedback loop between WAVE3 and PI3K-TGF-β-EGF signaling that plays a major role in BC development and progression.

## Materials and methods

### Cell lines and reagents

BC cell lines were obtained from American Type Culture Collection and maintained according to the manufacturer’s protocols. Cell lines were also routinely authenticated by short tandem repeat DNA fingerprinting analysis. WAVE3-KO cells were generated by lentiviral transduction as described previously^34^. We used two different and verified WAVE3-specific single guide RNAs (sgRNAs) for each of the human and mouse BC cell lines and a scrambled (SCRAM) sgRNA^34^. We used the following reagents: platelet-derived growth factor (PDGF; Millipore) (100 ng/ml for 10 min); TFG-β (R&D; 5 ng/ml for 20 min); EGF (Gibco) (100 ng/ml for 10 min); LY294002, ZD1839, SB431542, LY2109761, and Alpelisib (Selleckchem); AG1296 (Apexbio Technology); AKT1/2 Kinase-inhibitor (Sigma-Cat.# A6730; 10 µM for 4 h); and Matrigel (Corning; 4.5 mg/ml).

### Antibodies

We used the following antibodies: rabbit anti pAKT-S473, pAKT-T308, AKT, pERK1/2, ERK1/2, WAVE3 (Cell Signaling Technology); rabbit anti pSMAD3 and SMAD3 (Abcam); mouse anti-GFP (Clontech) (1:1000); goat horseradish-peroxidase-conjugated anti-mouse IgG and goat horseradish-peroxidase-conjugated anti-rabbit IgG (Calbiochem) (1:2000); mouse monoclonal anti-Actin (Sigma) (1:5000); and Alexa-Fluor-Plus-594-conjugated anti-rabbit IgG and Alexa-Fluor-Plus-488-conjugated anti-mouse IgG (Invitrogen). Vecta-shield with DAPI was from Vector Laboratories. Gel electrophoresis reagents were from Bio-Rad.

### Co-immunoprecipitation and western blot analyses

Protein cell lysate, co-immunoprecipitation, and western blot analyses were performed according to standards protocols as previously described^34,39^.

### Cell growth and migration assays

Cell growth was analyzed by IncuCyte Live-Cell Analysis System. Briefly, 2 × 10^4^ cells were seeded into 12-well culture plates with the complete culture media and scanned for 4 days. Cell migration was determined by wound-healing assay as previously described^34,40,41^. Wound closure was quantified by measuring wound areas from ≥3 different fields using ImageJ 1.8.0 (NIH).

### Three-dimensional tumorsphere cultures

#### 3D spheroid invasion

BC cell lines were seeded 2000 cells/well in 96-well ULA plate and centrifuged (125 × *g*,10 min) at room temperature. For single spheroid formation, each well was supplemented with 90 µl Matrigel in the complete culture media after 3 days. The plate was then placed into IncuCyte Live-Cell Analysis System and scheduled for a 6-h repeat scanning for 10 days.

#### 3D multi-spheroid formation

Ninety-six-well ULA plate was coated with 40 µl Matrigel with the complete culture media and allowed to polymerize at 37 °C for 30 min. Next, cells were seeded at a density of 1500 cells/well in 150 µl complete culture media. The plate was then incubated inside the IncuCyte Live-Cell Analysis System and scheduled for a 6-h repeat scanning for 10 days.

### Primary tumor growth

Parental (green fluorescent protein (GFP)), WAVE3-deficient (W3-KO), WAVE3-deficient overexpressing wild-type WAVE3 (W3-WT), or phospho-mutant WAVE3 (W3-Y4) MDA-MB-231 cells (10^6^ cells per mouse, *n* = 5) or 4T1 cells (10,000 cells per mouse, *n* = 5) were implanted into the mammary fat pads of 6–8-week-old female NSG or Balb/C mice, respectively. Tumor growth was monitored by twice weekly measuring tumor volume with digital Vernier calipers. At the endpoint, tumors were collected and fixed with 4% paraformaldehyde. Tumors were also weighed prior to fixation. All animal studies were performed under protocols approved by the Institutional Animal Care and Use Committee.

### Expression vectors and transfections

GFP-tagged WAVE3 constructs were generated as previously described^36^. The GFP-recombinant vector or the empty GFP expression control vector was used for stable transfections using standard protocols^34^. Oligonucleotide primers used for site-directed mutagenesis and sequencing were from Qiagen, were previously reported^36^, and are available upon request.

### Immunofluorescence of tumor tissues and confocal microscopy

BC tumor tissues were collected at the indicated times, snap-frozen in optimal cutting temperature medium (Sakura Finetek), and 8-μm sections were prepared and stained with the following antibodies: rabbit anti pAKT-S473 (1:400), pSMAD3(1:200), pERK1/2 (1:200), AKT (1:400), SMAD3 (1:500), and ERK1/2 (1:400), as described previously^22,39,42,43^. Stained sections were analyzed using fluorescence imaging microscopy (Leica) and ImagePro Plus Capture and Analysis software (Media Cybernetics). Positive areas were quantified in 15 independent fields/section using the Image Pro-Plus software^22,39,42,43^.

### Statistical analysis

Experiments were done in triplicate and analyzed using Student’s *t* test. In calculating two-tailed significance levels for equality of means, equal variances were assumed for the two populations. Results were considered significant at *p* < 0.05.

## Results

### WAVE3 tyrosine phosphorylation in BC cells is mediated by PDGF downstream of PI3K/AKT

**Fig. 1:**
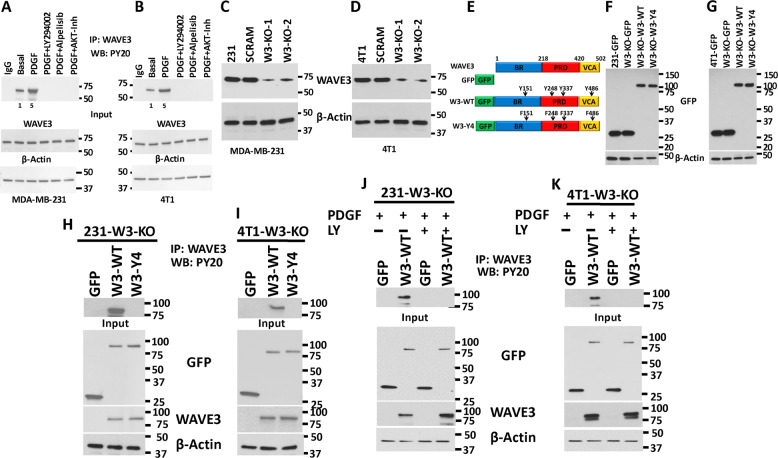
WAVE3 tyrosine phosphorylation in breast cancer cells is mediated by PDGF. **a**, **b** Protein lysates of MDA-MB-231 (**a**) and 4T1 (**b**) cells that were treated with either PDGF (100 ng/ml for 10 min) or with both PDGF (100 ng/ml for 10 min) and LY294002 (10 µM for 4 h) were immunoprecipitated with anti-WAVE3 antibody and subjected to immunoblotting with anti-phospho-tyrosine PY20. Rabbit IgG was used as a negative control (top panels). The numbers under the bands represent the fold change of the western blot signal normalized to the basal lane. The presence of equal amounts of WAVE3 and proteins in the cell lysates was confirmed by immunoblotting with anti-WAVE3 (input, middle panel). **c**, **d** Western blots developed with anti-WAVE3 antibody of lysates from control MDA-MB-231 (**c**) and 4T1 (**d**) transduced with a scrambled sgRNA (SCRAM) or two different WAVE3-specific sgRNAs (KO1 and KO2). **e** Graphic representation of the WAVE3 functional domains and GFP-fused truncation mutants. The location of the four tyrosine residues is also shown. BR basic region, PRD proline-rich domain, VCA verprolin, coffilin, and acidic. **f**, **g** Protein lysates of MDA-MB-231 (**f**) or 4T1 (**g**) cells that were induced to express the indicated phenotype were developed with anti-GFP antibody. **h**, **i** Proteins lysate prepared from WAVE3-deficient MDA-MB-231 (**h**) or 4T1 (**i**) cells that were transfected with GFP, wild-type (W3-WT), or phospho-mutant WAVE3 (W3-Y4) were used for immunoprecipitation with anti-WAVE3 antibody and subjected to immunoblotting analysis with anti-PY20 antibody. Cell lysates were also immunoblotted with anti-GFP and anti-WAVE3 antibodies to show expression of the fusion proteins and the presence of equal amounts of these proteins in the cell lysates (input panels). **j**, **k** (input panels) In all cases, the size of proteins identified on the western blot is as expected for the protein fragment. β-Actin is a loading control.

Our previous studies have shown that WAVE3 is phosphorylated downstream of PI3K^27,36^. However, those findings were based on in vitro experiments performed in Cos7 cells^36^. Given the overwhelming evidence supporting WAVE3 as a critical driver of the invasion–metastasis cascade in BC (reviewed in ref. ^23^), the data established in Cos7 cells did not reflect the potential involvement of WAVE3 phosphorylation in the pathology of BC. To this end, first we showed that basal levels of tyrosine phosphorylation of endogenous WAVE3 are detected in both MDA-MB-231 and 4T1 BC cells (Fig. 1a, b). Phosphorylation levels of WAVE3 increased by fivefold (*p* < 0.05) after PDGF treatment, and this phosphorylation is completely lost after treatment with the PDGFR inhibitor LY294002, PI3K inhibitor, Alpelisib, or with an AKT1/2 kinase inhibitor (Fig. 1a, b). Thus we confirm that WAVE3 tyrosine phosphorylation is mediated downstream of PI3K/AKT signaling. Our published studies also showed that 4 tyrosine residues (Y151, Y248, Y337, and Y486, Fig. 1e) are phosphorylated^36^. To investigate the role of WAVE3 phosphorylation at these specific tyrosine residues, we used CRISPR/Cas9 to generate pools of WAVE3-knokout cells^34^ in MDA-MB-231 (Fig. 1c) and 4T1 (Fig. 1d) cells. WAVE3-knockout derivatives were then transduced to stably express GFP alone (W3-KO-GFP), wild-type WAVE3-GFP fusion (W3-KO-W3-WT), or a phospho-mutant WAVE3-GFP fusion (W3-KO-W3-Y4), where all four tyrosine residues (Fig. 1e) were mutated to unphosphorylated phenylalanine^36^. Western blot analysis was used to confirm the expression of GFP and the WAVE3-GFP fusion proteins in both MDA-MB-231 (Fig. 1f) and 4T1 (Fig. 1g) cell lines and their WAVE3-KO derivatives. Protein lysates from these cells were subjected to immunoprecipitation with anti-WAVE3 antibody, followed by western blot of the resulting immunocomplexes with anti-phosphotyrosine antibody (PY20) to detect phosphorylation levels of WAVE3. In the PDGF-treated MDA-MB-231 (Fig. 1h) and 4T1 (Fig. 1i) cells, W3-WT but not phospho-mutant WAVE3 (W3-Y4) can be detected by PY20 antibody in the WAVE3 immunocomplexes, indicating that mutation of the four tyrosine residues resulted in complete loss of WAVE3 phosphorylation. To confirm that PDGF (PI3K signaling) is indeed required for the activation of WAVE3 phosphorylation, W3-KO MDA-MB-231 (Fig. 1j) or 4T1 (Fig. 1k) cells that were transduced to express either GFP or wild-type WAVE3 were stimulated with PDGF in combination with or without the PI3K inhibitor LY294002. The cell lysates were then subjected to co-immunoprecipitation analyses. As expected, W3-WT showed a strong phosphorylation in the presence of PDGF, while treatment with LY294002 blunted this phosphorylation (Fig. 1h, i). Thus these data confirm the involvement of the four tyrosine residues in the PDGF-mediated phosphorylation of WAVE3.

### WAVE3 phosphorylation is required for migration and invasion of BC cells in vitro

**Fig. 2:**
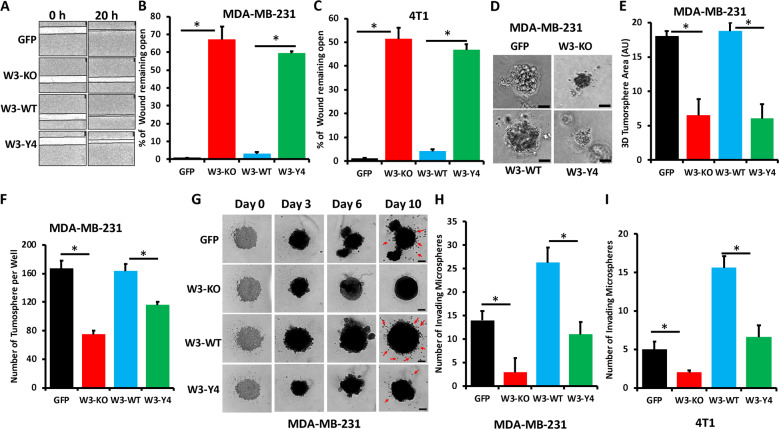
WAVE3 phosphorylation is required for migration and invasion of BC cells in vitro. **a** Representative micrographs of wound healing assays of confluent cell cultures of parental MDA-MB-231 cells (GFP), their WAVE3-deficient derivatives (W3-KO), or the W3-KO expressing either wild-type (W3-WT) or phospho-mutant (W3-Y4) WAVE3 that were induced to migrate into scratch wounds in confluent monolayers over 20 h. The unclosed wound (open area) at 20 h from 12 different wounds was measured and plotted as the percentage of the wound at time zero for MDA-MB-231 cells (**a**, **b**) and 4T1 cells (**c**). **d** Representative micrographs of parental MDA-MB-231 and its derivatives induced to form tumorspheres. **e**, **f** Quantification of surface area occupied by resulting tumorspheres (**e**) and the number of tumorspheres formed (**f**). **g** Representative micrographs of parental MDA-MB-231 and its derivatives induced to form tumorspheres. At day 3, Matrigel was added to the tumorsphere cultures and cells that invaded the Matrigel (arrows) were quantified at day 10 both for MDA-MB-MB-231 (**h**) and 4T1 cells (**i**). Data are the mean ± SD (*n* = 3, *p < 0.05; Student’s *t* test).

The role of WAVE3 in cell migration and invasion of cancer cells of multiple origins is well established^27–31,36,44–46^. Whether WAVE3 phosphorylation plays an important role in such oncogenic activities has not been reported. Parental MDA-MB-231 or 4T1 or their W3-KO derivatives expressing GFP, wild-type WAVE3, or phospho-mutant WAVE3 were subjected to a two-dimensional wound healing, and the extent of the wound that remained open at the end of the experiment was compared between the experimental groups. First, it is important to note that loss of WAVE3 expression or re-expression of wild-type or phospho-mutant WAVE3 did not affect cell proliferation of both MDA-MB-231 (Fig. S1A) and 4T1 cells (Fig. S1B). Therefore, differences in wound closure are the results of differences in cell migration but not differences in cell proliferation. In MDA-MB-231 cells, almost 70% of wound remained open in the W3-KO cells, compared to the parental cells (Fig. 2a, b), confirming the previously published role of WAVE3 in cell migration^25,27,28,33,44^. In the W3-KO cells expressing W3-WT, the wound was almost completely closed, while re-expression of W3-Y4 achieved <60% of wound closure (Fig. 2a, b). This finding was duplicated in 4T1 cells and its derivatives (Fig. 2c), thus confirming the role of WAVE3 phosphorylation in cell migration of BC cells. We also used three-dimensional (3D) tumorsphere growth and invasion assays to assess the role of phosphorylated WAVE3 in cancer cell growth and invasion in 3D conditions. We found loss of WAVE3 (W3-KO) in MDA-MB-231 cells to significantly (*p* < 0.05) inhibit the growth (Fig. 2d, e) and number (Fig. 2f) of tumorspheres, compared to the parental cells (GFP). However, while re-expression of W3-WT in the W3-KO cells was able to restore the growth of the tumorspheres, re-expression of W3-Y4 did not (Fig. 2d–f). Additionally, we found loss of WAVE3 expression to significantly inhibit the Matrigel invasion of MDA-MB-231 (Fig. 2g, h) and 4T1 (Fig. 2i). We also found that re-expression of W3-WT but not that of W3-Y4 restored 3D cell invasion (Fig. 2g–i). Therefore, our findings confirm the involvement of tyrosine phosphorylation in the in vitro oncogenic activities of WAVE3 in BC cells.

### WAVE3 phosphorylation is required for tumor growth of BC cells in vivo

**Fig. 3:**
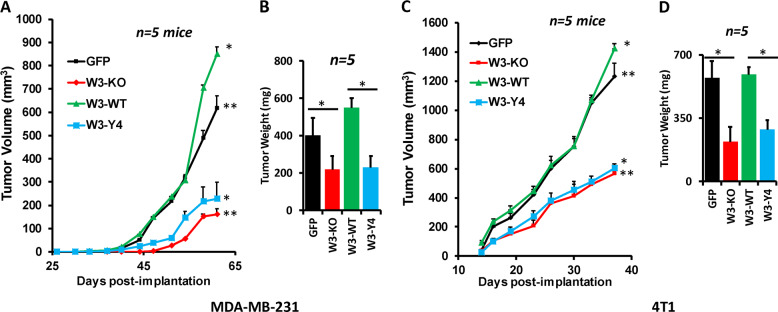
WAVE3 phosphorylation is required for tumor growth of BC cells in vivo. Quantification of volume (**a**, **c**) and weight (**b**, **d**) of tumors generated from inoculation of parental MDA-MB-231 (**a**, **b**) and 4T1 cells (**c**, **d**) and their derivatives into the mammary fat pads of NSG mice for MDA-MB-231 and Balb/c mice for 4T1 cells. Data are generated from five mice per group. Data are the mean ± SD (*n* = 5, *^,^**p < 0.05; Student’s *t* test).

To assess the effects of loss of WAVE3 phosphorylation on tumor growth in vivo, mammary fat pads of NSG mice were inoculated with parental MDA-MB-231 cells (GFP), W3-KO, or W3-KO re-expressing either W3-WT or W3-Y4, and tumor growth was assessed over 8 weeks. Loss of WAVE3 inhibited the growth of primary tumors (Fig. 3a). Re-expression W3-WT not only restored tumor growth but also enhanced tumor growth to levels that were significantly higher (*p* < 0.05) than those obtained with the parental group, while re-expression of W3-Y4 resulted in tumor growth similar to that obtained with the W3-KO group (Fig. 3b). While every mouse in every group developed tumors (100% tumor incidence) after a ∼4-week latency, tumor burden, as assessed by tumor volume (Fig. 3a) and weight (Fig. 3b), was significantly lower (*p* < 0.05) in the mice implanted with the WAVE3-deficient cells and the WAVE3-deficient re-expressing phospho-mutant WAVE3. Similarly, loss of murine WAVE3 and re-expression of W3-Y4 in the W3-KO 4T1 cells also delayed tumor initiation and growth in the BALB/c mice (Fig. 3c), while re-expression of the W3-WT fully restored tumor growth (Fig. 3b). Thus loss of WAVE3 phosphorylation inhibits the rate of primary tumor growth in vivo in both human and mouse models for TNBC. These differences in tumor burden were not a result of decreased tumor cell proliferation by manipulation of WAVE3 expression of posttranscriptional modification, as the number of viable cells between the control and WAVE3-deficient cells was similar (Fig. S1A, B).

### WAVE3 phosphorylation is also mediated downstream of TGF-β and EGF signaling pathways

**Fig. 4:**
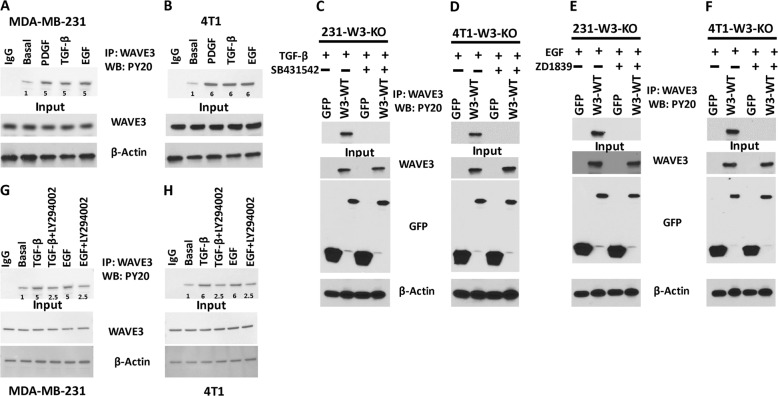
WAVE3 phosphorylation is also mediated downstream of TGF-β and EGF signaling pathways. Protein lysates of MDA-MB-231 (**a**) and 4T1 (**b**) cells were treated with PDGF (100 ng/ml for 10 min), TGF-β (5 ng/ml for 20 min), or EGF (100 ng/ml for 10 min); immunoprecipitated with anti-WAVE3 antibody; and subjected to immunoblotting with anti-phospho-tyrosine PY20. Rabbit IgG was used as a negative control (top panels). The presence of equal amounts of WAVE3 and proteins in the cell lysates was confirmed by immunoblotting with anti-WAVE3 (input, middle panel). **c**, **d** Protein lysate prepared from WAVE3-deficient MDA-MB-231 (**c**) or 4T1 (**d**) cells that were transfected with GFP or W3-WT and treated with TGF-β in the presence or absence of SB431542 were used for immunoprecipitation with anti-WAVE3 antibody and subjected to immunoblotting analysis with anti-PY20 antibody. **e**, **f** Proteins lysate prepared from WAVE3-deficient MDA-MB-231 (**e**) or 4T1 (**f**) cells that were transfected with GFP or W3-WT and treated with EGF in the presence or absence of ZD1839 were used for immunoprecipitation with anti-WAVE3 antibody and subjected to immunoblotting analysis with anti-PY20 antibody. For **c**–**f**, cell lysates were also immunoblotted with anti-GFP and anti-WAVE3 antibodies to show expression of the fusion proteins and the presence of equal amounts of these proteins in the cell lysates (input panels). In all cases, the size of proteins identified on the western blot are as expected for the protein fragment. **g**, **h** MDA-MB-231 (**g**) and 4T1 (**h**) cells were treated with PDGF (100 ng/ml for 10 min), TGF-β (5 ng/ml for 20 min), or EGF (100 ng/ml for 10 min). TGF-β- and EGF-stimulated cells were also treated with the PI3K inhibitor LY294002 (10 µM for 4 h). Protein lysates were then immunoprecipitated with anti-WAVE3 antibody and subjected to immunoblotting with anti-phospho-tyrosine PY20. Rabbit IgG was used as a negative control (top panels). The numbers under the bands represent the fold change of the western blot signal normalized to the basal lane. The presence of equal amounts of WAVE3 and proteins in the cell lysates was confirmed by immunoblotting with anti-WAVE3 (input, middle panel). β-Actin is a loading control.

Our published data showed that WAVE3 tyrosine phosphorylation can be mediated downstream of PI3K^27,36^. We showed that the oncogenic activity of WAVE3 is enhanced downstream of TGF-β^32^. Whether WAVE3 phosphorylation, which is associated with its oncogenic activity, is also mediated downstream of TGF-β or EGF, has, however, not been reported. We therefore sought to investigate whether TGF-β is involved in WAVE3 phosphorylation. Stimulation of cultured cells with PDGF, TGF-β, or EGF increased phosphorylation levels of WAVE3 in both MDA-MB-231 (Fig. 4a) and 4T1 cells (Fig. 4b) by at least fivefold (p < 0.05), compared to basal levels. To demonstrate that WAVE3 phosphorylation is a direct effect of the activation of either TGF-β or EGF signaling pathways, we found that treatment with TGF-β (Fig. 4c, d) or EGF (Fig. 4e, f) inhibitors (SB431542 and ZD1839, respectively) inhibited WAVE3 phosphorylation in both MDA-MB-231 (Fig. 4c, e) and 4T1 (Fig. 4d, f) cells. In addition, treatment with the PI3K inhibitor LY294002 after stimulation with either TGF-β or EGF resulted in ~3-fold decrease (*p* < 0.05) but not complete loss of WAVE3 phosphorylation, when compared to phosphorylation levels after stimulation with either TGF-β or EGF without the inhibitor (Fig. 4g, h). We also confirmed that these signaling pathways are functional in the cell lines used in this study (Fig. S2). PDGF-mediated phosphorylation of AKT, TGF-β-mediated phosphorylation of SMAD3, and the EGF-mediated phosphorylation of ERK1/2 were almost completely abolished after treatment with the respective inhibitors of these signaling pathways (Figs. S3 and S4). Treatment with the PI3K inhibitor LY294002 after stimulation with either TGF-β or EGF did not result in a complete inhibition of the TGF-β- or EGF-mediated phosphorylation of WAVE3 (Fig. 4g, h). This suggests that, in addition to PI3K, WAVE3 phosphorylation can be activated downstream of other oncogenic signaling pathways, e.g., TGF-β and EGF, and that the WAVE3 phosphorylation downstream of PI3K can also be regulated by TGF-β and EGF signaling, potentially independent of PI3K. Along these lines, we found that PDGF-mediated phosphorylation of AKT can be slightly inhibited in the presence of TGF-β signaling inhibitor (SB431542) or EGF signaling inhibitor (ZD1839) (Fig. S5A, B), further supporting our findings.

### WAVE3 phosphorylation regulates the interplay between WAVE3 and PI3K, TGF-β, and EGF signaling pathways

**Fig. 5:**
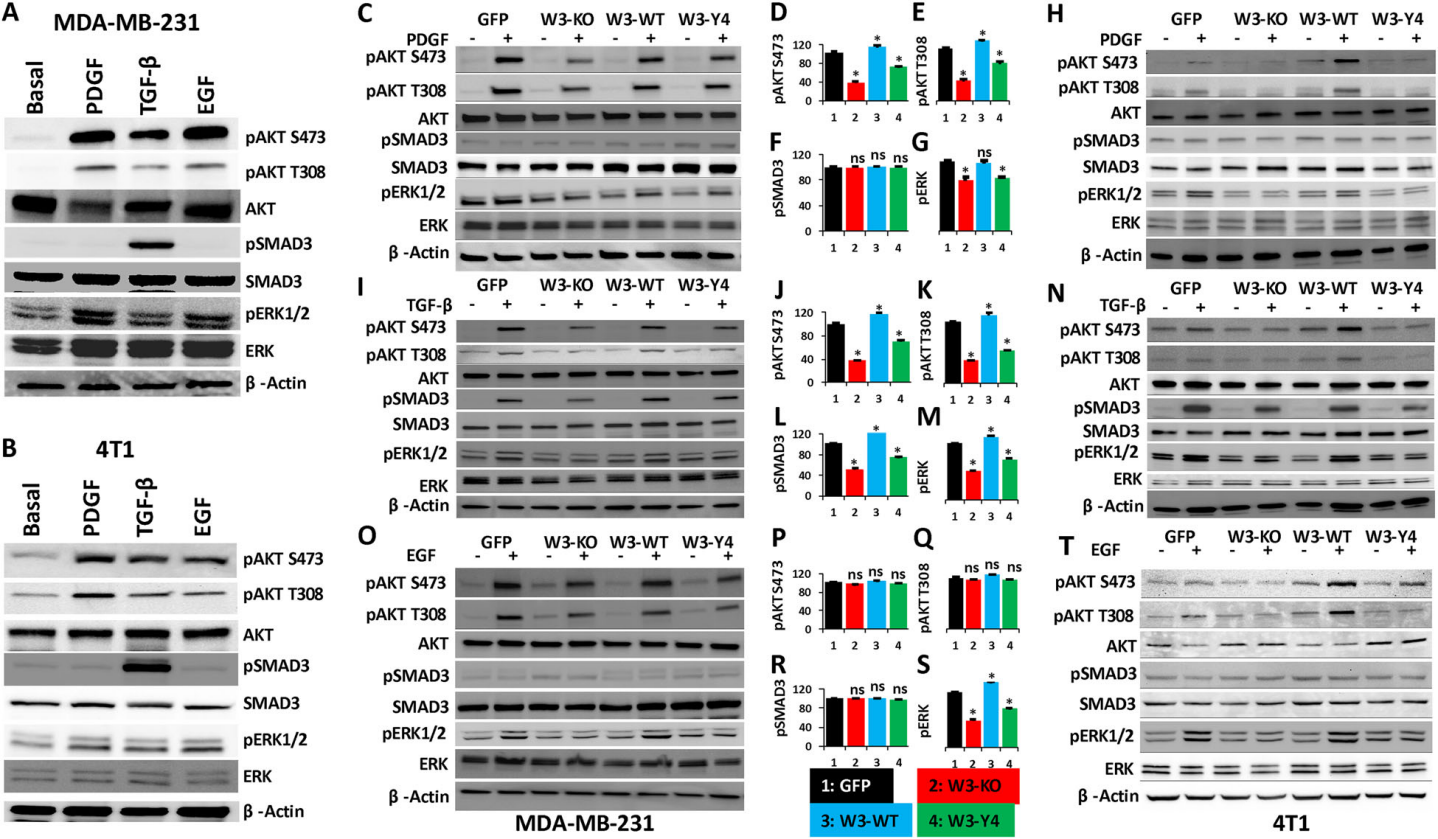
WAVE3 phosphorylation regulates the interplay between WAVE3 and PI3K, TGF-β, and EGF signaling pathways in vitro. **a**, **b** Protein lysates of MDA-MB-231 (**a**) and 4T1 (**b**) cells were treated as indicated and subjected to immunoblotting with antibodies against the indicated phospho proteins and their total counterparts. β-Actin is a loading control. **c**–**h** Protein lysates of untreated (−) or PDGF-stimulated (+) MDA-MB-231 (**c**) and 4T1 cells (**h**) were subjected to immunoblotting with antibodies against the indicated phospho proteins and their total counterparts. **d**–**g** Quantification of phosphorylation levels in MDA-MB-231 of pAKT S473 (**d**), pAKT T308 (**e**), pSMAD3 (**f**), and pERK1/2 (**g**). **i**–**n** Protein lysates of untreated (−) or TGF-β-stimulated (+) MDA-MB-231 (**i**) and 4T1 cells (**n**) were subjected to immunoblotting with antibodies against the indicated phospho proteins and their total counterparts. **j**–**m** Quantification of phosphorylation levels in MDA-MB-231 of pAKT S473 (**j**), pAKT T308 (**k**), pSMAD3 (**l**), and pERK1/2 (**m**). **o**–**t** Protein lysates of untreated (−) or EGF-stimulated (+) MDA-MB-231 (**o**) and 4T1 cells (**t**) were subjected to immunoblotting with antibodies against the indicated phospho proteins and their total counterparts. **p**–**s** Quantification of phosphorylation levels in MDA-MB-231 of pAKT S473 (**p**), pAKT T308 (**q**), pSMAD3 (**r**), and pERK1/2 (**s**). Cell lysates were also immunoblotted with antibodies against total AKT, SMAD3, and ERK1/2 to demonstrate expression of equal amounts of these proteins in the cell lysates. β-Actin is a loading control. Densitometric analyses were used to quantify signal intensity and changes in phosphorylation levels were plotted in percentage change to GFP after normalization to the untreated control and are average values from three different blots. Data are the mean ± SD, *N* = 3 independent replicates; ns not significant; *p < 0.05; Student’s *t* test).

Crosstalk between oncogenic signals is a well-established mechanism during the activation of the invasion–metastasis cascade in cancer tumors. To better understand how WAVE3 phosphorylation regulates its oncogenic activity downstream of PI3K, TGF-β, and EGF, we investigated the effect of loss of WAVE3 phosphorylation on the activation of these signaling pathways. First, we confirmed that all three signaling pathways are active in both MDA-MB-231 and 4T1 cells. PDGF, TGF-β1, and EGF treatment of MDA-MB-231 cells (Fig. 5a) and 4T1 cells (Fig. 5b) stimulated the PI3K, TGF-β, and EGF downstream effectors AKT, SMAD3, and ERK1/2, respectively, as demonstrated by increase in their phosphorylation levels. Additionally, we found that TGF-β treatment of MDA-MB-231 (Fig. 5a) and 4T1 cells (Fig. 5b) also increased phosphorylation of both AKT and ERK1/2, while PDGF or EGF treatment did not enhance phosphorylation levels of SMAD3 beyond basal levels. Thus TGF-β signaling may be active upstream of both PI3K and ERK1/2. On the other hand, PDGF or EGF treatment also increased phosphorylation of both AKT and ERK1/2 but not that of SMAD3, indicating that, while the PI3K (AKT) and EGF (ERK1/2) can be activated downstream of TGF-β, they can also be interchangeably activated by their respective stimulants, e.g., PDGF and EGF, respectively (Fig. 5a, b). This observation, while it may have already been reported in the literature, is important for the interpretation of the data that follows.

Next, to investigate the molecular mechanisms involving WAVE3 in the regulation of PI3K, TGF-β, and EGF oncogenic signaling pathways, cell cultures of parental (GFP) MDA-MB-231 and 4T1 cells, W3-KO, and their W3-WT- and W3-Y4-expressing derivatives were treated with PDGF (Fig. 5c, h), TGF-β (Fig. 5i, n), or EGF (Fig. 5o, v), and phosphorylation levels of their downstream effectors were assessed by western blot and compared to their untreated counterparts. Parental MDA-MB-231 and 4T1 cells (GFP) were used as controls. As expected, PDGF treatment increased AKT phosphorylation of both S473 and T308 in the parental MDA-MB-231 (Fig. 5c–e) and 4T1 cells (Fig. 5h). Interestingly, in the W3-KO cells phosphorylation levels of AKT were at least twofold less (*p* < 0.05) than those seen in the parental cells. Re-expression of W3-WT increased AKT phosphorylation levels >20% (*p* < 0.05) compared to those seen in the parental cells, while re-expression of W3-Y4 failed to do so. AKT phosphorylation levels in W3-Y4 were comparable to those seen in the W3-KO, with at least a twofold decrease (*p* < 0.05) compared to parental controls. Thus WAVE3 phosphorylation is required for the PDGF-mediated activation of PI3K and its downstream effector AKT. Likewise, PDGF treatment increased phosphorylation levels of pERK1/2 in parental MDA-MB-231 (Fig. 5c, g) and 4T1 cells (Fig. 5h). Re-expression of W3-WT increased pERK1/2 levels by ~20% (*p* < 0.05), while both W3-KO and re-expression of W3-Y4 prevented the PDGF-mediated phosphorylation of ERK1/2 (~30% decrease, *p* < 0.05), compared to parental cells (Fig. 5c, g, h). On the other hand, no significant changes in phosphorylation of pSMAD3 were observed after PDGF treatment in all the experimental groups (Fig. 5c, f, h), suggesting that manipulation of WAVE3 expression or its phosphorylation status may have no effect on PI3K signaling to modulate SMAD phosphorylation levels. LY294002 treatment after PDGF stimulation inhibited AKT phosphorylation in all four groups (Fig. S6A, B). We also found that TGF-β treatment efficiently enhanced phosphorylation of its direct downstream effector SMAD3 as well as that of AKT and ERK1/2 in the parental MDA-MB-231 (Fig. 5i–m) and 4T1 (Fig. 5n) cells. Conversely, phosphorylation levels of SMAD3, AKT, and ERK1/2 in W3-KO cells were decreased by at least threefold (*p* < 0.05) compared to the parental cells, and re-expression of wild-type WAVE3 in the WAVE3-deficient cells resulted in phopho-SMAD3, -AKT, and -ERK1/2 levels comparable to the parental cells, while re-expression of phospho-mutant WAVE3 failed to restore SMAD3 phosphorylation beyond basal levels (Fig. 5i–n). SB431542 treatment after TGF-β stimulation inhibited phosphorylation of AKT and ERK1/2, in addition to SMAD3 (Fig. S7A, B). These data strongly suggest that (i) TGF-β is an upstream activator of both PI3K and EGF signaling and (ii) WAVE3 phosphorylation is required for the regulation of TGF-β-mediated activation of both PI3K and EGF signaling. Similar trend was observed in the EGF treatment, where EGF-mediated phosphorylation of ERK1/2 was inhibited by at least threefold (*p* < 0.05) in both the WAVE3-deficient MDA-MB-231 (Fig. 5o, s) and 4T1 cells (Fig. 5t) as well as in their phospho-mutant-expressing derivatives. EGF treatment did not, however, affect phosphorylation levels of either AKT or SMAD, suggesting that EGF signaling may not function upstream of PI3K and TGF-β signaling (Fig. 5o–t), while treatment with ZD1839 inhibited the EGF-mediated phosphorylation of ERK1/2 (Fig. S8A, B). Therefore, these data confirm the requirement of WAVE3 phosphorylation for the activation of the PI3K, TGF-β, and EGF downstream signaling and strongly support the interplay between WAVE3 and these signaling pathways in the activation of WAVE3 oncogenic function in BC.

### WAVE3 phosphorylation is required for the PI3K-, TGF-β-, and EGF-mediated activation of tumorsphere formation and invasion

**Fig. 6:**
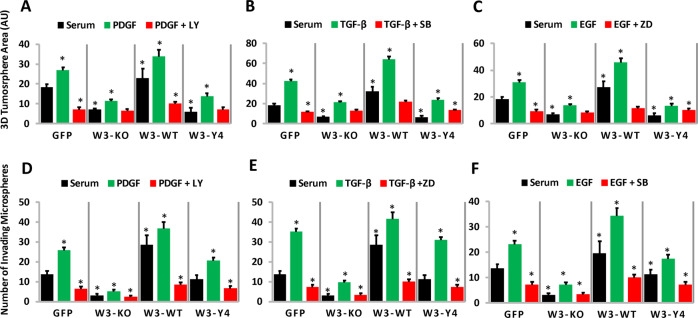
WAVE3 phosphorylation is required for the PI3K-, TGF-β-, and EGF-mediated activation of tumorsphere formation and invasion. Quantification of tumorsphere surface area (**a**–**c**) and tumorsphere invasion (**d**–**f**) of MDA-MB-231 and its derivatives, under the described conditions. PDGF (100 ng/m/), TGF-β (5 ng/ml), or EGF (100 ng/ml) and their respective inhibitors (10 µM) were added to the culture every 48 h. For tumorsphere invasion, Matrigel was added at day 3 to the tumorsphere cultures, and cells that invaded the Matrigel were quantified at day 10. Data are the mean ± SD (*n* = 3, *p < 0.05; Student’s *t* test).

The biological significance of the interplay between WAVE3 phosphorylation and the PI3K, TGF-β, and EGF signaling pathways is further demonstrated by the ability of WAVE3 phosphorylation to regulate the 3D tumorsphere growth (Fig. 6a–c) and invasion of the extracellular matrix (Fig. 6d–f), downstream of these signaling pathways. In the serum-treated cells, tumorsphere growth (Fig. 6a–c black bars) and invasion (Fig. 6d–f, black bars) is significantly higher (*p* < 0.05) in the GFP and the W3-WT cells, compared to their W3-KO and the W3-Y4 counterparts. Treatment with PDGF (Fig. 6a, d), TGF- β (Fig. 6b, e), or EGF (Fig. 6c, f) resulted in a significant (*p* < 0.05) increase in tumorsphere size (Fig. 6a–c, green bars) and invasion (Fig. 6d–f, green bars) in the GFP and the W3-WT cells, compared to their W3-KO and the W3-Y4 counterparts. Inhibition of the PDGF, TGF-β, and EGF signaling by their respective inhibitors blunted tumorsphere growth (Fig. 6a–c, red bars) and invasion (Fig. 6d–f, red bars) in all four groups, albeit inhibition of tumorsphere growth was much prominent in the W3-KO and W3-Y4 cells than in the GFP and W3-WT cells. These results suggest that (i) WAVE3 expression and its phosphorylation are required for tumorsphere growth, which is even more enhanced in the presence of PDGF, TGF-β, or EGF and (ii) loss of WAVE3 expression or its phosphorylation status inhibit the PDGF-, TGF-β- or EGF-mediated activation of tumorsphere growth, further supporting the positive feedback loop between WAVE3 in these signaling pathways.

**Fig. 7:**
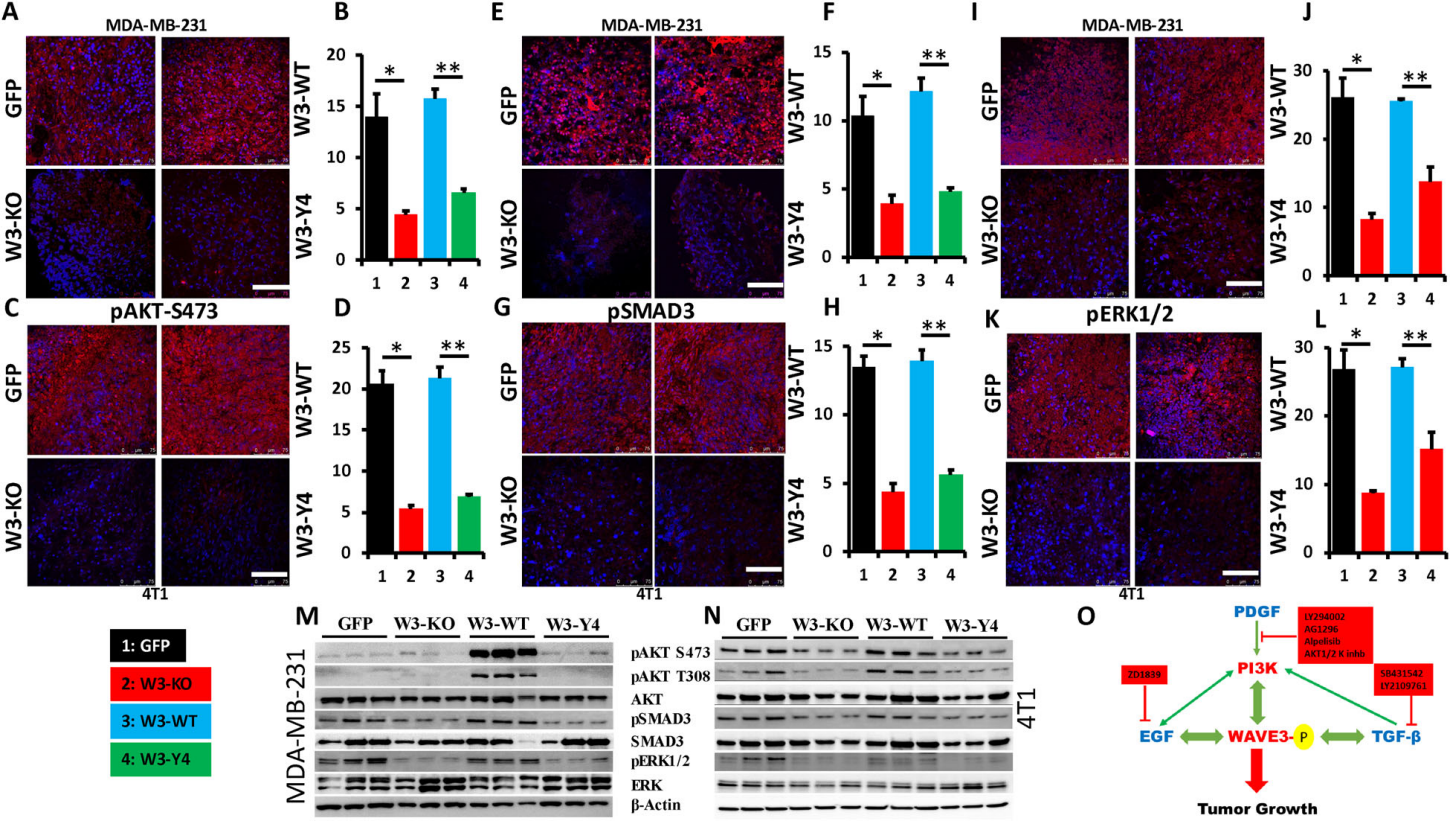
WAVE3 phosphorylation regulates the interplay between WAVE3 and PI3K, TGF-β, and EGF signaling pathways in vivo. Representative confocal microscopic images of tumor sections stained for pAKT-S473 (red) of MDA-MB-231 (**a**) and 4T1 (**b**) and their derivatives. Size bar, 75 µm. Cell nuclei were counterstained with DAPI (blue). **b**, **d** Quantification of pAKT-S473-positive areas in MDA-MB-231 (**b**) and 4T1 (**d**) tumors. Data are expressed as mean ± SEM. *^,^**p < 0.001, *n* = 5 mice. **e**, **g** Representative confocal microscopic images of tumor sections stained for pSMAD3 (Red) of MDA-MB-231 (**e**) and 4T1 (**g**) and their derivatives. Cell nuclei were counterstained with DAPI (blue). Size bar, 75 µm. **f**, **h** Quantification of pSMAD3-positive areas in MDA-MB-231 (**f**) and 4T1 (**h**) tumors. Data are expressed as mean ± SEM. *^,^**p < 0.001, *n* = 5 mice. **i**, **k** Representative confocal microscopic images of tumor sections stained for pERK1/2 (Red) of MDA-MB-231 (**i**) and 4T1 (**k**) and their derivatives. Cell nuclei were counterstained with DAPI (blue). Size bar, 75 µm. **j**, **l** Quantification of pERK1/2-positive areas in MDA-MB-231 (**j**) and 4T1 (**l**) tumors. Data are expressed as mean ± SEM. *^,^**p < 0.001, *n* = 5 mice. **m**, **n** Protein lysates from tumors derived from the parental (GFP) MDA-MB-231 (**m**) and 4T1 (**n**) cells, and their derivatives were analyzed by immunoblotting with the indicated antibodies. β-Actin was used as loading control. **o** Model describing the interplay between WAVE3, its phosphorylation, and the PI3K/TGF-β/EGF signaling pathways in regulation of tumor growth in BC.

Our findings were further confirmed in vivo in the tumors derived from MDA-MB-231 and 4T1 cells (GFP), their W3-KO derivatives, and from the WAVE3-deficient MDA-MB-231 and 4T1 cells re-expressing W3-WT or W3-Y4. We used immunohistochemistry to stain sections of the tumors described above with antibodies against phospho-AKT S473, phospho-SMAD3, and phospho-ERK1/2. The staining revealed a significant decrease (*p* < 0.05) in phosphorylation levels of AKT in the tumor derived from the W3-KO tumors as well as from the tumors derived from W3-Y4, compared to the tumors derived from the parental cells (GFP) or the tumors derived from W3-WT, both for MDA-MB-231 (Fig. 7a, b) and 4T1 cells (Fig. 7c, d). A similar trend was observed for the phosphorylation levels of SMAD3 (Fig. 7e–h) and ERK1/2 (Fig. 7i–l); levels of phospho-SMAD3 and phospho-ERK1/2 were inhibited as a result of WAVE3-KO or lack of its phosphorylation and were restored by re-expression of wild-type WAVE3 but not of phospho-mutant WAVE3. Finally, we used western blot analyses with total protein lysates of the tumor tissues to show a significant increase in phosphorylation levels of AKT, SMAD3, and ERK1/2 in tumors derived from the MDA-MB-231 (Fig. 7m) and 4T1 cells (Fig. 7n) re-expressing W3-WT but not W3-Y4. Phospho-AKT and phospho-SMAD levels were at least tenfold higher (*p* < 0.05) in the tumors derived from W3-WT cells and in those derived from their W3-Y4 counterparts, both in the MDA-MB-231 (Fig. 7m) and the 4T1 (Fig. 7n) groups. Therefore, our findings are supported by both in vitro as well as in vivo investigations.

## Discussion

This study showed that WAVE3 phosphorylation supports tumor growth in BC and does so at least in part through its activation downstream of PI3K/TGF-β/EGF oncogenic signaling pathways. WAVE3 has been established as a major driver of the invasion–metastasis cascade in BC as well as other tumors. Increased expression levels of WAVE3 correlates with tumor growth, invasion, and metastasis^32,33^ and with poor outcome in cancer patients^34,44,47,48^. The oncogenic activity of WAVE3 is associated with several hallmarks of cancer^23^, by regulating the EMT program, tumor angiogenesis, and the CSC niche^28,34,44^. Most of the studies investigating the function of WAVE3 in cancer progression and metastasis have, however, focused on changes in the expression levels of WAVE3 at the protein and mRNA levels. Few other studies have also described the regulation of WAVE3 activity through posttranscriptional modification. We have shown that the oncogenic activity of WAVE3 can be inhibited through the posttranscriptional targeting of its mRNA by microRNA miR200 and miR31^33,49^. Our previous studies identified a new pathway for the regulation of WAVE3 activity at the posttranslational level^36^. WAVE3 phosphorylation at specific tyrosine residues downstream of PI3K was found to be critical for the WAVE3-mediated regulation of cell migration^27,36^. Whether WAVE3 phosphorylation is also required for its oncogenic activity in driving tumor growth and progression has, however, not been reported. To our knowledge, this is the first study to show that WAVE3 phosphorylation can be mediated not only by PI3K, as previously reported, but also downstream of TGF-β and EGF, thereby providing a novel mechanism by which increased WAVE3 phosphorylation correlates with BC tumor growth and invasion. In addition, we showed that WAVE3 phosphorylation is involved in a positive feedback signaling loop that activates PI3K, TGF-β, and EGF signaling.

We applied a combination of genetic and pharmacologic manipulations, as well as different biochemical and cell imaging analyses in vitro and in mouse models, to investigate the role of WAVE3 phosphorylation in the regulation of the growth and progression of BC tumors. Our investigations used CRISPR/Cas9 gene editing to delete WAVE3 in human and murine BC cell lines^22,34,39,43^. We also used lentivirus-mediated infections^34,39^ to induce expression of either wild-type or phospho-mutant form of WAVE3 in the WAVE3-deficient BC cells. We showed that (i) WAVE3 tyrosine phosphorylation is mediated not only downstream of PI3K but TGF-β and EGF can also and equally induce WAVE3 phosphorylation; (ii) in vitro, WAVE3 phosphorylation is required for BC cell migration and 3D tumorsphere growth and invasion; (iii) in in vivo mouse models, WAVE3 is required for tumor growth, and the extent of tumor growth depends on WAVE3 phosphorylation; and (iv) WAVE3 phosphorylation is involved in the interplay between WAVE3 and PI3K, TGF-β, and EGF signaling pathways: while PI3K, TGF-β, and EGF stimulate phosphorylation of WAVE3, increased WAVE3 phosphorylation, in turn, activates the signaling of PI3K, TGF-β, and EGF. Thus we have established a positive feedback loop where WAVE3/PI3K/TGF-β/EGF signaling axis plays a key role in the regulation of tumor growth in BC (Fig. 7o).

While the PI3K-mediated activation of WAVE3 phosphorylation was previously discussed^36^, the molecular mechanism by which TGF-β and EGF stimulate WAVE3 phosphorylation is, however, not known. PI3K was found to activate WAVE3 phosphorylation through the binding of its regulatory p85α subunit to WAVE3, therefore allowing the recruitment of the non-receptor tyrosine kinase cAbl, which, in turn, phosphorylates WAVE3 at specific tyrosine residues (Fig. 1e, refs. ^27,36^). As for TGF-β, in addition to SMAD-mediated canonical TGF-β signaling, the TGF-β receptors can also activate other intracellular pathways, either through phosphorylation of or through direct interaction with critical signaling intermediates. These SMAD-independent or non-canonical TGF-β signaling pathways comprise several branches, including the PI3K/AKT pathway, the EGF/ERK1/2^10,50^; and their TGF-β-mediated activation may be a potential molecular mechanism through which WAVE3 is phosphorylated downstream of TGF-β and EGF. One SMAD-dependent TGF-β molecular signaling was described, where the TGF-β-induced activation of PI3K was mediated through the ubiquitin ligase TRAF6 and a SMAD protein^15,16^. Upon TGF-β stimulation, PI3K is recruited and its regulatory subunit p85α is polyubiquitylated by TRAF6, resulting in the phosphorylation of the PI3K catalytic subunit p110 and the activation of its downstream effectors^15^, including WAVE3 phosphorylation. A second SMAD-independent TGF-β mechanism was described through a direct interaction between PI3K and the TGF-β type I receptor^17^. Another potential molecular mechanism by which WAVE3 regulates the PI3K/AKT pathway in this positive feedback loop may be through the WAVE3-mediated activation of PDK2 (pyruvate dehydrogenase kinase isoform 2), which in turn activates AKT phosphorylation^38^, resulting in the promotion of cell proliferation, migration, and invasion of cancer cells, as well the activation of EMT program and the CSC phenotype^35,38^. Our data also showed that WAVE3 phosphorylation can be mediated downstream of TGF-β and EGF, possibly independent of PI3K, since treatment with PI3K inhibitors of TGF-β- and EGF-stimulated cells did not completely abrogate WAVE3 phosphorylation (Fig. 4g, h).

Whether all four tyrosine residues are required for the oncogenic activity of WAVE3 remains to be addressed. Our published studies identified that the proline-rich domain (PRD) of WAVE3 is required for the maintenance of the CSC phenotype in TNBC^34^. PRD is also the host of two of the four tyrosine residues responsible of the oncogenic activity of WAVE3. The fact that WAVE3 was found to be associated with several hallmarks of cancer^19,23^ was tempting to speculate that the other two tyrosine residues located outside the PRD domain in the basic region (BR) and the VCA domain, respectively (Fig. 1e) may be involved in other WAVE3-associated oncogenic activities. We do recognize the complexity of the interactions between WAVE3 and PI3K, TGF-β, and EGF signaling pathways and how these interactions drive the oncogenic properties of WAVE3. While in-depth investigation of the molecular mechanisms that regulate these interactions is of paramount significance, it is beyond the scope of the present study and will be addressed in future investigations. In conclusion, our study established a novel WAVE3/PI3K/TGF-β/EGF signaling axis in BC and suggest that inhibition of WAVE3 activity may be a BC therapeutic target.

## Supplementary information

Supplemental Figures

Legends to Supplemental Figures
