# Supplementary material for: WAVE3 phosphorylation regulates the interplay between PI3K, TGF-β, and EGF signaling pathways in breast cancer
Source: Oncogenesis. 2020 Oct 5;9(10):87. doi: 10.1038/s41389-020-00272-0 (PMC7533250; doi:10.1038/s41389-020-00272-0)
Supplement: Supplementary file 1 — Supplemental Figures [file 41389_2020_272_MOESM1_ESM.pdf]

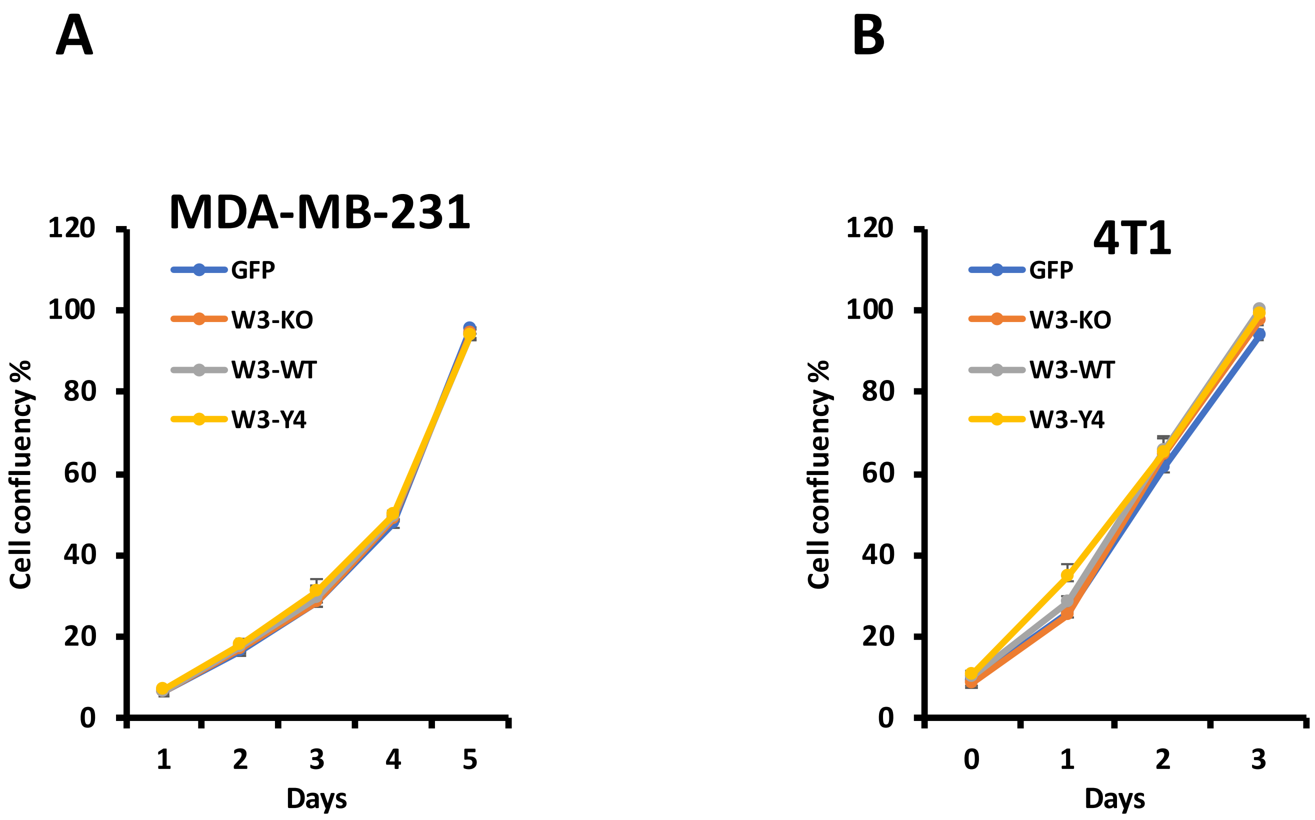
A
B
MDA-MB-231
4T1
GFP
W3-KO
W3-WT
W3-Y4
Cell confluency %
Days
120
100
80
60
40
20
0
1
2
3
4
5
0
1
2
3

Figure S1

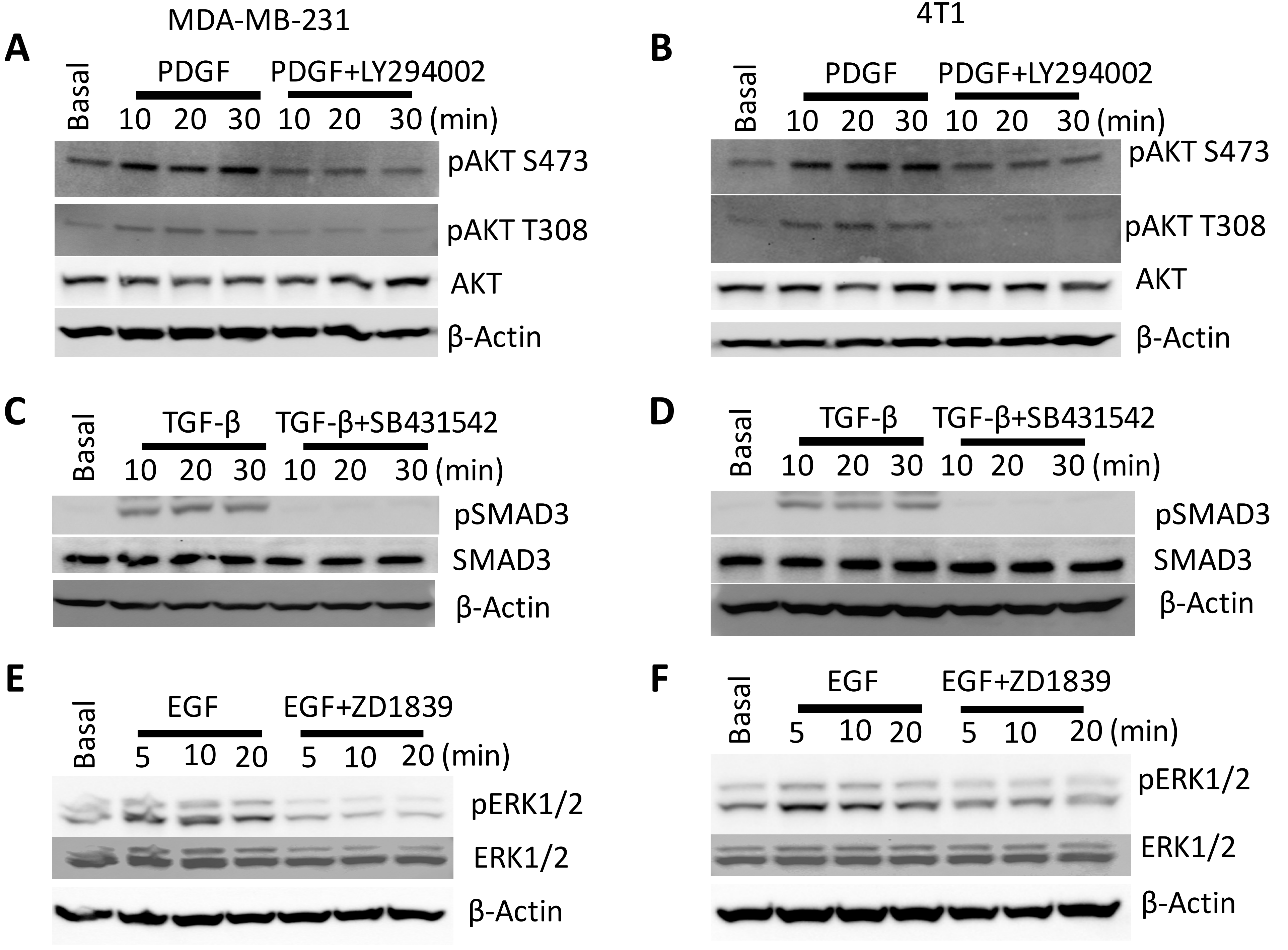
MDA-MB-231
4T1
A
Basal
PDGF
PDGF+LY294002
10 20 30 10 20 30 (min)
pAKT S473
pAKT T308
AKT
β-Actin
B
Basal
PDGF
PDGF+LY294002
10 20 30 10 20 30 (min)
pAKT S473
pAKT T308
AKT
β-Actin
C
Basal
TGF-β
TGF-β+SB431542
10 20 30 10 20 30 (min)
pSMAD3
SMAD3
β-Actin
D
Basal
TGF-β
TGF-β+SB431542
10 20 30 10 20 30 (min)
pSMAD3
SMAD3
β-Actin
E
Basal
EGF
EGF+ZD1839
5 10 20 5 10 20 (min)
pERK1/2
ERK1/2
β-Actin
F
Basal
EGF
EGF+ZD1839
5 10 20 5 10 20 (min)
pERK1/2
ERK1/2
β-Actin

Figure S2

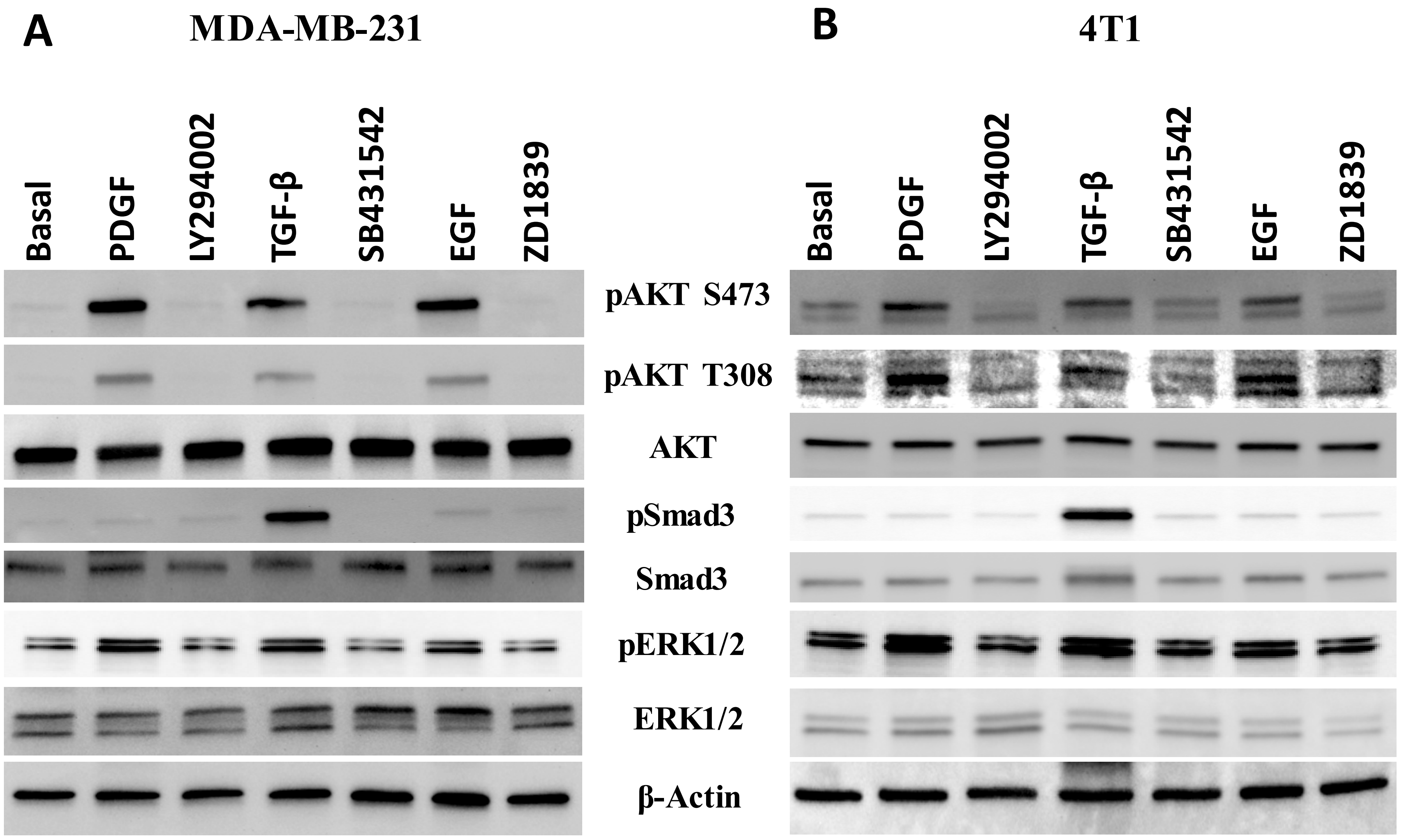
A
MDA-MB-231
B
4T1
Basal
PDGF
LY294002
TGF-β
SB431542
EGF
ZD1839
pAKT S473
pAKT T308
AKT
pSmad3
Smad3
pERK1/2
ERK1/2
β-Actin

Figure S3

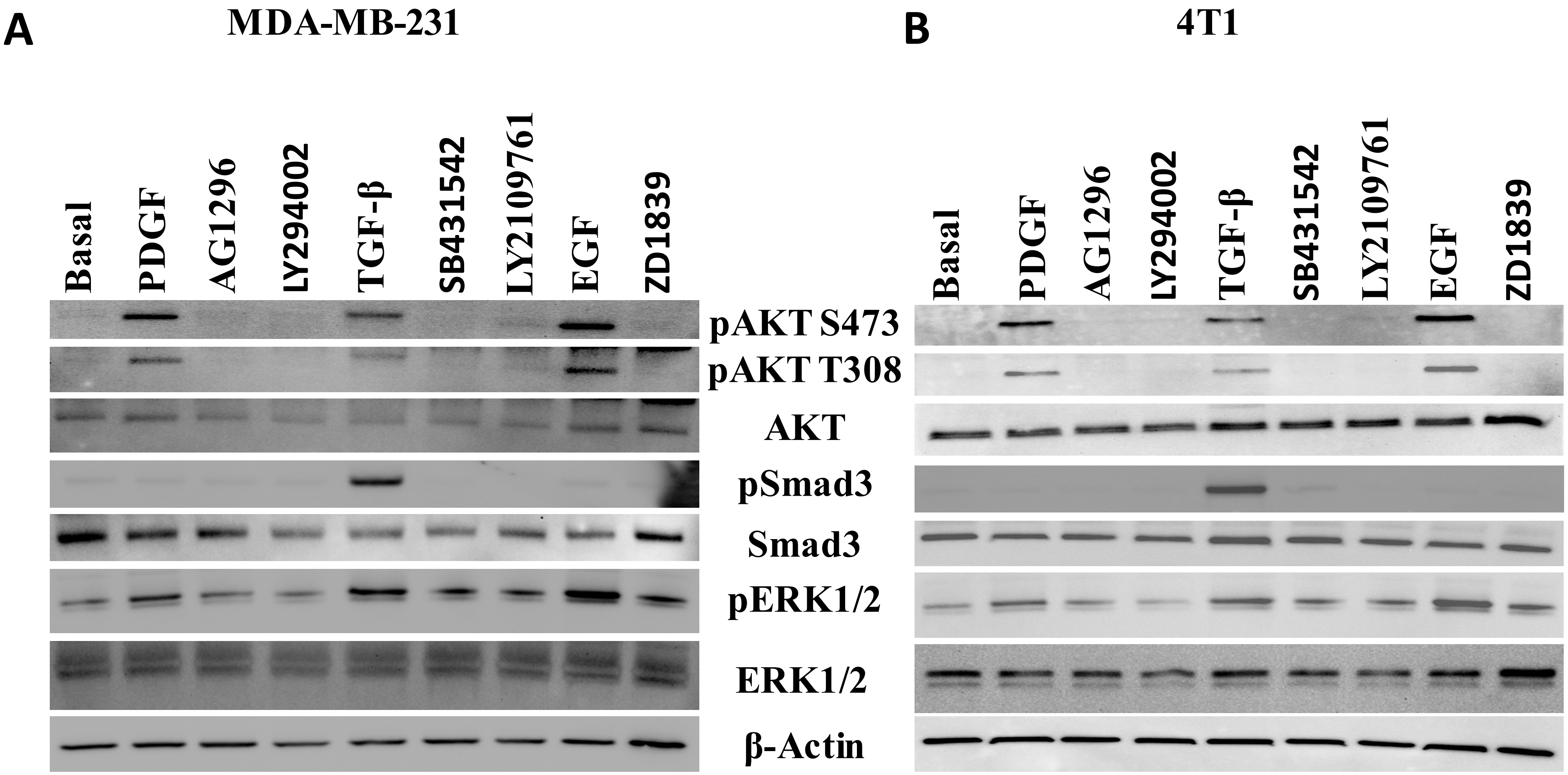
A
MDA-MB-231
B
4T1
Basal
PDGF
AG1296
LY294002
TGF-β
SB431542
LY2109761
EGF
ZD1839
pAKT S473
pAKT T308
AKT
pSmad3
Smad3
pERK1/2
ERK1/2
β-Actin

Figure S4

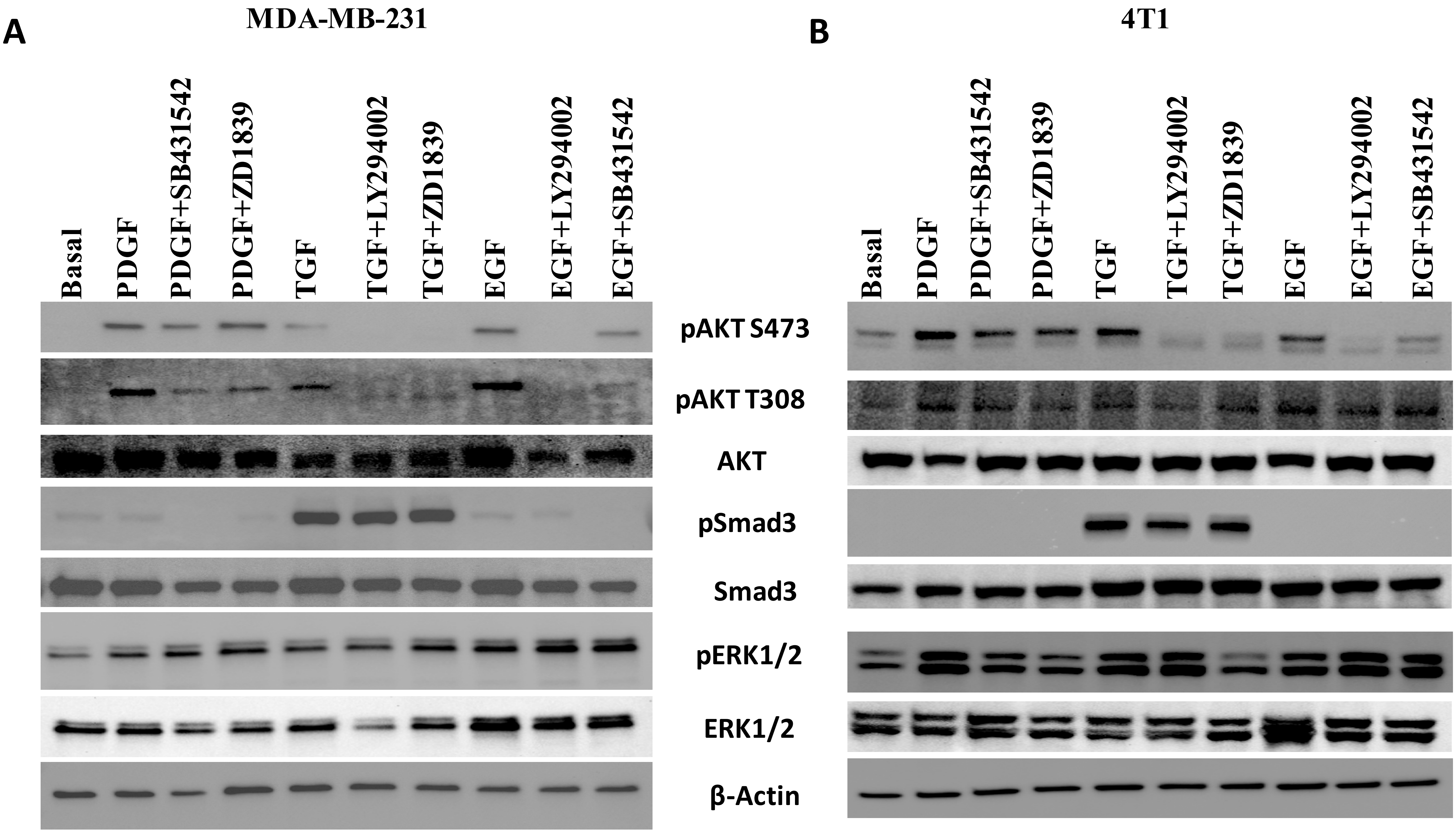
A
MDA-MB-231
B
4T1
Basal
PDGF
PDGF+SB431542
PDGF+ZD1839
TGF
TGF+LY294002
TGF+ZD1839
EGF
EGF+LY294002
EGF+SB431542
pAKT S473
pAKT T308
AKT
pSmad3
Smad3
pERK1/2
ERK1/2
β-Actin

Figure S5

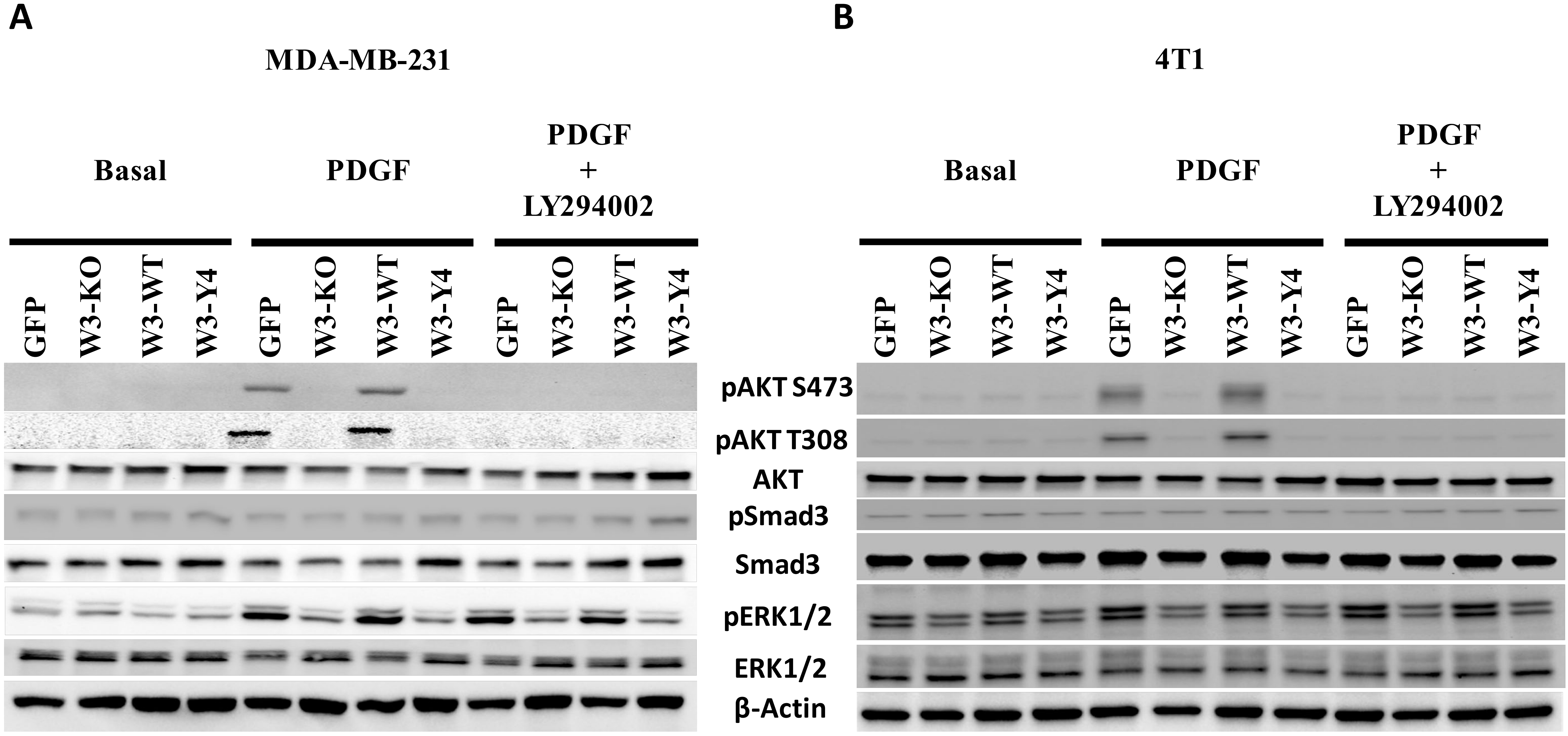
A
MDA-MB-231
Basal
PDGF
PDGF + LY294002
GFP
W3-KO
W3-WT
W3-Y4
B
4T1
pAKT S473
pAKT T308
AKT
pSmad3
Smad3
pERK1/2
ERK1/2
β-Actin

Figure S6

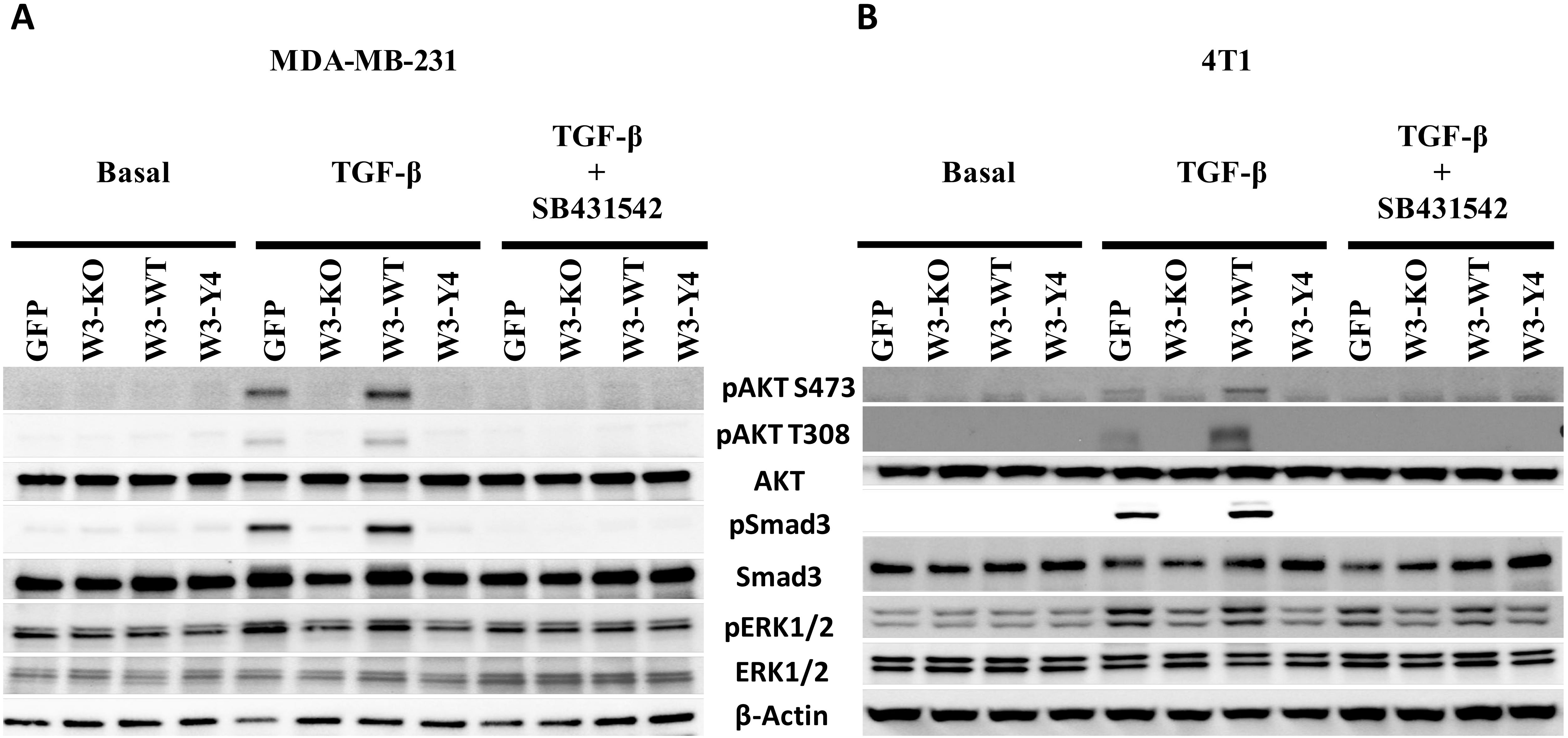
A
MDA-MB-231
Basal
TGF-β
TGF-β + SB431542
GFP
W3-KO
W3-WT
W3-Y4
B
4T1
pAKT S473
pAKT T308
AKT
pSmad3
Smad3
pERK1/2
ERK1/2
β-Actin

Figure S7

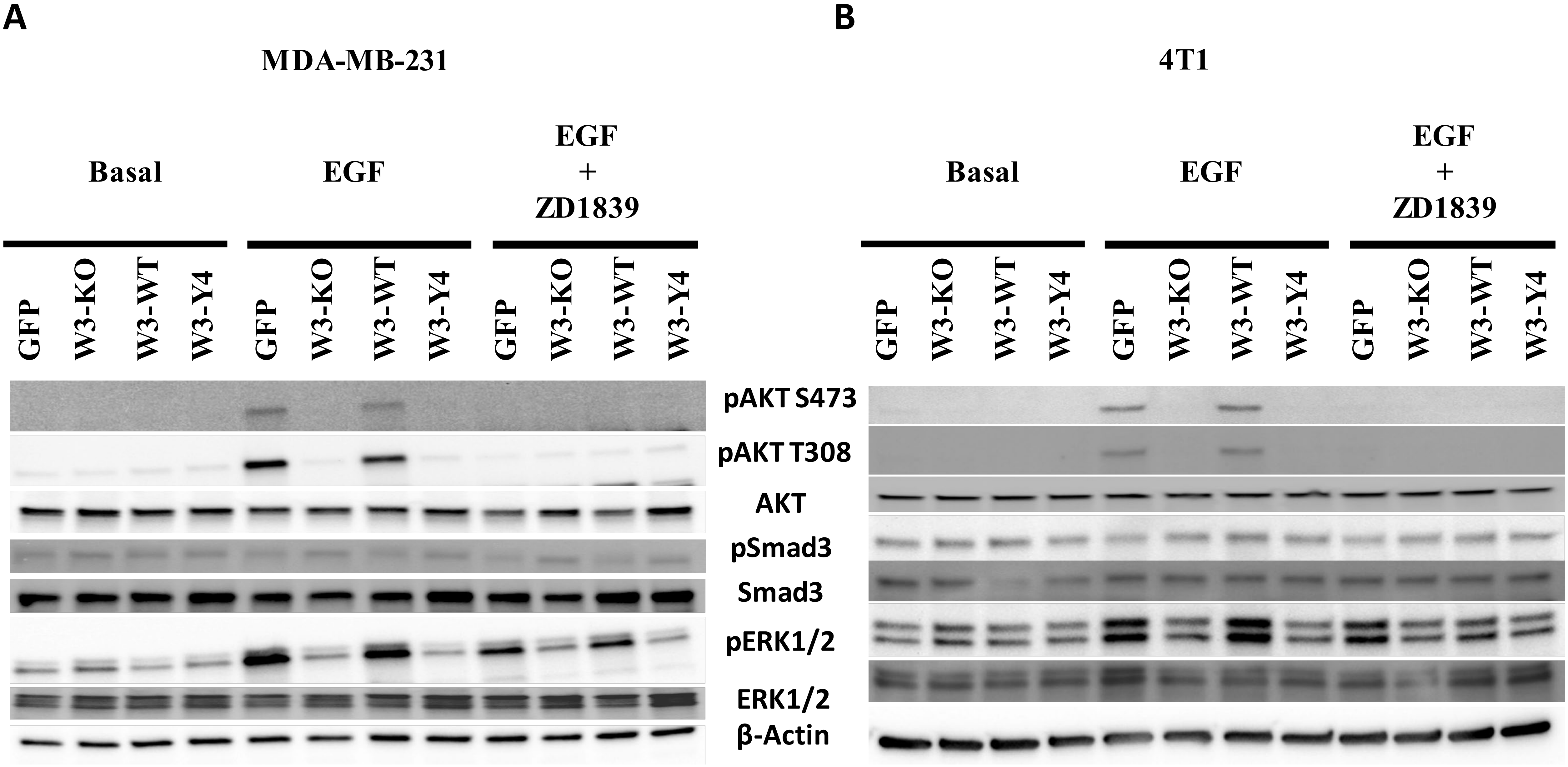
A
MDA-MB-231
Basal
EGF
EGF + ZD1839
GFP
W3-KO
W3-WT
W3-Y4
B
4T1
pAKT S473
pAKT T308
AKT
pSmad3
Smad3
pERK1/2
ERK1/2
β-Actin

Figure S8
