# Supplementary material for: WAVE3 phosphorylation regulates the interplay between PI3K, TGF-β, and EGF signaling pathways in breast cancer
Source: Oncogenesis. 2020 Oct 5;9(10):87. doi: 10.1038/s41389-020-00272-0 (PMC7533250; doi:10.1038/s41389-020-00272-0)
Supplement: Supplementary file 2 — Legends to Supplemental Figures [file 41389_2020_272_MOESM2_ESM.docx]

**Supplemental Figures Legends**

**Figure S1**. Cell proliferation over 5 days of parental MDA-MB-231 (S1A) and 4T1 (S1B) cells (GFP), their WAVE3-deficient derivatives (W3-KO), the W3-KO cells expressing wild-type (W3-WT) or phosphomutant (W3-Y4) WAVE3. Data are the means ± SD from 3 replicate experiments.

**Figure S2**. (A and B) MDA-MB-231 (A) and 4T1 (B) cells were stimulated with PDGF (100 ng/ml) for the indicated times or treated with LY294002(10 µM) for 4 hr. after being stimulated with PDGF (100 ng/ml) for the indicated times. (C and D) MDA-MB-231 (C) and 4T1 (D) cells were stimulated with TGF-β (5 ng/ml) for the indicated times or treated with SB431542 (10 µM) for 4 hr. after being stimulated with TGF-β (5 ng/ml) for the indicated times. (E and F) MDA-MB-231 (E) and 4T1 (F) cells were stimulated with EGF (100 ng/ml) for the indicated times or treated with ZD1839 (10 µM) for 4 hr. after being stimulated with EGF (100 ng/ml) for the indicated times. The Basal lane refers to unstimulated and untreated cells. The resulting protein lysates subjected to immunoblotting with antibodies against the indicated phospho proteins and their total counterparts. β-Actin is a loading control.

**Figure S3**. MDA-MB-231 (A) and 4T1 (B) cells were stimulated with PDGF (100 ng/ml) for 10 min or treated with Ly294002 (10 µM) for 4 hr. after being stimulated with PDGF (100 ng/ml) for 10 min. Cells were also stimulated with TGF-β (5 ng/ml) for 20 min. or treated with SB431542 (10 µM) for 4 hr. after being stimulated with TGF-β (5 ng/ml) for 20 min. Cells were stimulated with EGF (100 ng/ml) for 10 min. or treated with ZD1839 (10 µM) for 4 hr. after being stimulated with EGF (100 ng/ml) for 10 min. The Basal lane refers to unstimulated and untreated cells. The resulting protein lysates subjected to immunoblotting with antibodies against the indicated phospho proteins and their total counterparts. β-Actin is a loading control.

**Figure S4**. MDA-MB-231 (A) and 4T1 (B) cells were stimulated with PDGF (100 ng/ml) for 10 min or treated with Ly294002 (10 µM) for 4 hr. after being stimulated with PDGF (100 ng/ml) for 10 min, or treated with AG1296 (10 µM) for 4 hr. after being stimulated with PDGF (100 ng/ml) for 10 min. Cells were also stimulated with TGF-β (5 ng/ml) for 20 min. or treated with SB431542 (10 µM) for 4 hr. after being stimulated with TGF-β (5 ng/ml) for 20 min, or treated with LY2109761 (10 µM) for 4 hr. after being stimulated with TGF-β (5 ng/ml) for 20 min. Cells were also stimulated with EGF (100 ng/ml) for 10 min. or treated with ZD1839 (10 µM) for 4 hr. after being stimulated with EGF (100 ng/ml) for 10 min. `The Basal lane refers to unstimulated and untreated cells. The resulting protein lysates subjected to immunoblotting with antibodies against the indicated phospho proteins and their total counterparts. β-Actin is a loading control.

**Figure S5**. MDA-MB-231 (A) and 4T1 (B) cells were stimulated with PDGF (100 ng/ml) for 10 min or treated with SB431542 (10 µM) for 4 hr. after being stimulated with PDGF (100 ng/ml) for 10 min, or treated with ZD1839 (10 µM) for 4 hr. after being stimulated with PDGF (100 ng/ml) for 10 min. Cells were also stimulated with TGF-β (5 ng/ml) for 20 min. or treated with LY294002 (10 µM) for 4 hr. after being stimulated with TGF-β (5 ng/ml) for 20 min, or treated with ZD1839 (10 µM) for 4 hr. after being stimulated with TGF-β (5 ng/ml) for 20 min. Cells were also stimulated with EGF (100 ng/ml) for 10 min. or treated with LY294002 (10 µM) for 4 hr. after being stimulated with EGF (100 ng/ml) for 10 min., or treated with LY294002 (10 µM) for 4 hr. after being stimulated with EGF (100 ng/ml) for 10 min. `The Basal lane refers to unstimulated and untreated cells. The resulting protein lysates subjected to immunoblotting with antibodies against the indicated phospho proteins and their total counterparts. β-Actin is a loading control.

**Figure S6**. Protein lysates of untreated (Basal) or PDGF-stimulated or PDGF-stimulated and LY294002-treated MDA-MB-231 (A) and 4T1 cells (B) or their derivatives, were subjected to immunoblotting with antibodies against the indicated phospho proteins and their total counterparts. β-Actin is a loading control. GFP: Control cells; W3-KO: WAVE3-deficient cells; W3-WT: WAVE3-deficient cells overexpressing wildtype WAVE3; W3-Y4: WAVE3-deficient cells overexpressing phosphomutant WAVE3.

**Figure S7**. Protein lysates of untreated (Basal) or TGF-β-stimulated or TGF-β-stimulated and SB431542-treated MDA-MB-231 (A) and 4T1 cells (B) or their derivatives, were subjected to immunoblotting with antibodies against the indicated phospho proteins and their total counterparts. β-Actin is a loading control. GFP: Control cells; W3-KO: WAVE3-deficient cells; W3-WT: WAVE3-deficient cells overexpressing wildtype WAVE3; W3-Y4: WAVE3-deficient cells overexpressing phosphomutant WAVE3.

**Figure S8**. Protein lysates of untreated (Basal) or EGF-stimulated or EGF-stimulated and ZD1839-treated MDA-MB-231 (A) and 4T1 cells (B) or their derivatives, were subjected to immunoblotting with antibodies against the indicated phospho proteins and their total counterparts. β-Actin is a loading control. GFP: Control cells; W3-KO: WAVE3-deficient cells; W3-WT: WAVE3-deficient cells overexpressing wildtype WAVE3; W3-Y4: WAVE3-deficient cells overexpressing phosphomutant WAVE3.
